# Impact of pretransplant T2DM on left ventricular deformation and myocardial perfusion in heart transplanted recipients: a 3.0 T cardiac magnetic resonance study

**DOI:** 10.1186/s12933-024-02323-x

**Published:** 2024-06-21

**Authors:** Liqi Cao, Chang Liu, Chulan Ou, Quanmei Ma, Huanwen Xu, Xiaodan Li, Yingying Bao, Rui Chen, Yuelong Yang, Min Wu, Hui Liu

**Affiliations:** 1 Department of Radiology, Guangdong Provincial People’s Hospital (Guangdong Academy of Medical Sciences), Southern Medical University, Guangzhou, 510080 China; 2 The Second School of Clinical Medicine, Southern Medical University, Guangzhou, China; 3 School of Medicine, South China University of Technology, Guangzhou, China; 4 The First Affiliate Hospital of Guangzhou Medical University, Guangzhou, China; 5 Deparment of Heart Transplantation and VAD surgery, Guangdong Cardiovascular Institute, Guangdong Provincial People’s Hospital, Guangdong Academy of Medical Sciences, Guangzhou, China; 6 Guangdong Provincial Key Laboratory of Artificial Intelligence in Medical Image Analysis and Application, Guangdong Provincial People’s Hospital, Guangdong Academy of Medical Sciences, Guangzhou, China

**Keywords:** Type 2 diabetes mellitus, Heart transplant, Cardiovascular magnetic resonance, Left ventricular strains, First-pass perfusion

## Abstract

**Background:**

Pretransplant type 2 diabetes mellitus (T2DM) is associated with increased cardiovascular and all-cause mortality after heart transplant (HT), but the underlying causes of this association remain unclear. The purpose of this research was to examine the impact of T2DM on left ventricular (LV) myocardial deformation and myocardial perfusion following heart transplantation using cardiovascular magnetic resonance imaging.

**Methods:**

We investigated thirty-one HT recipients with pretransplant T2DM [HT(DM+)], thirty-four HT recipients without pretransplant T2DM [HT(DM−)] and thirty-six controls. LV myocardial strains, including the global longitudinal, radial, and circumferential strain (GLS, GRS and GCS, respectively), were calculated and compared among groups, as were resting myocardial perfusion indices, which included time to peak myocardial signal intensity (TTM), maximum signal intensity (MaxSI), and Upslope. The relationships between LV strain parameters or perfusion indices and biochemical indicators were determined through Spearman’s analysis. The impact of T2DM on LV strains in HT recipients was assessed using multivariable linear regression analyses with backward stepwise selection.

**Results:**

In the HT(DM+) group, the LV GLS, GRS, and GCS exhibited significantly lower magnitudes than those in both the HT(DM−) and control groups. TTM was higher in the HT(DM+) group than in both the HT(DM−) and control groups, while no significant differences were observed among the groups regarding Upslope and MaxSI. There was a negative correlation between glycated hemoglobin and the magnitude of strains (longitudinal, r = − 0.399; radial, r = − 0.362; circumferential, r = − 0.389) (all P < 0.05), and a positive correlation with TTM (r = 0.485, P < 0.001). Regression analyses that included both pretransplant T2DM and perfusion indices revealed that pretransplant T2DM, rather than perfusion indices, was an independent determinant of LV strain (β = longitudinal, − 0.508; radial, − 0.370; circumferential, − 0.371) (all P < 0.05).

**Conclusion:**

In heart transplant recipients, pretransplant T2DM has a detrimental effect on subclinical left ventricular systolic function and could potentially impact myocardial microcirculation following HT.

**Supplementary Information:**

The online version contains supplementary material available at 10.1186/s12933-024-02323-x.

## Introduction

Type 2 diabetes mellitus (T2DM) is a prevalent comorbidity in patients with severe heart failure, affecting approximately 40% of individuals diagnosed with this condition [1, 2]. In recent years, driven by the encouraging post-transplant outcomes observed in T2DM patients without signs of end-organ damage, a growing number of T2DM patients have been listed for heart transplant (HT) and subsequently transplanted [3]. Despite the correction of cardiac dysfunction through transplantation, T2DM adversely impacts HT recipients’ outcomes by increasing infection risk, impeding wound healing, and increasing the risk of cardiovascular events [4]. A large-scale study of 38,004 HT recipients [5], including 9917 T2DM patients, demonstrated significantly higher all-cause mortality at 5 years post-HT among pretransplant T2DM patients than among non-T2DM patients (24.4% vs 20.6%). Accordingly, the latest guidelines from the International Society for Heart and Lung Transplantation (ISHLT) recommend that diabetes prevention, early identification, and proper treatment should be considered a crucial part of post-HT patient care [6].

Cardiometabolic abnormalities and chronic inflammation are widely recognized as key factors in the development of diabetes-related myocardial remodeling and dysfunction [7]. Previous research has provided evidence of early metabolic abnormalities in the transplanted healthy hearts of diabetic recipients, underscoring the association between abnormal lipid accumulation in cardiomyocytes and cardiac dysfunction in HT recipients with pretransplant T2DM [8]. Moreover, the presence of T2DM can exacerbate endothelial inflammation, thereby leading to endothelial dysfunction and microvascular rarefaction, ultimately resulting in microvascular dysfunction [9]. Therefore, promptly detecting myocardial dysfunction and impaired microvascular perfusion may be crucial in HT recipients with pretransplant T2DM to recommend tailored treatment strategies, aimed at mitigating the risk of cardiovascular complications and enhancing outcomes.

Cardiovascular magnetic resonance (CMR) has been shown to be a valuable imaging technique in the field of cardiology owing to its distinctive and intricate imaging methodologies, which can simultaneously assess cardiac anatomy and function along with myocardial perfusion in the same examination. Utilizing CMR imaging, it was observed that T2DM adversely affects cardiac systolic function and myocardial perfusion in both uncomplicated individuals and those with comorbidities such as hypertension [10, 11]. However, as far as we know, research on the impact of T2DM on heart function and microcirculation in HT recipients is scarce. In this study, our objective was to investigate the influence of pretransplant T2DM on left ventricular deformation and myocardial perfusion post-heart transplantation by employing CMR feature tracking (FT) and first-pass perfusion.

## Methods

### Study population and design

**Fig. 1:**
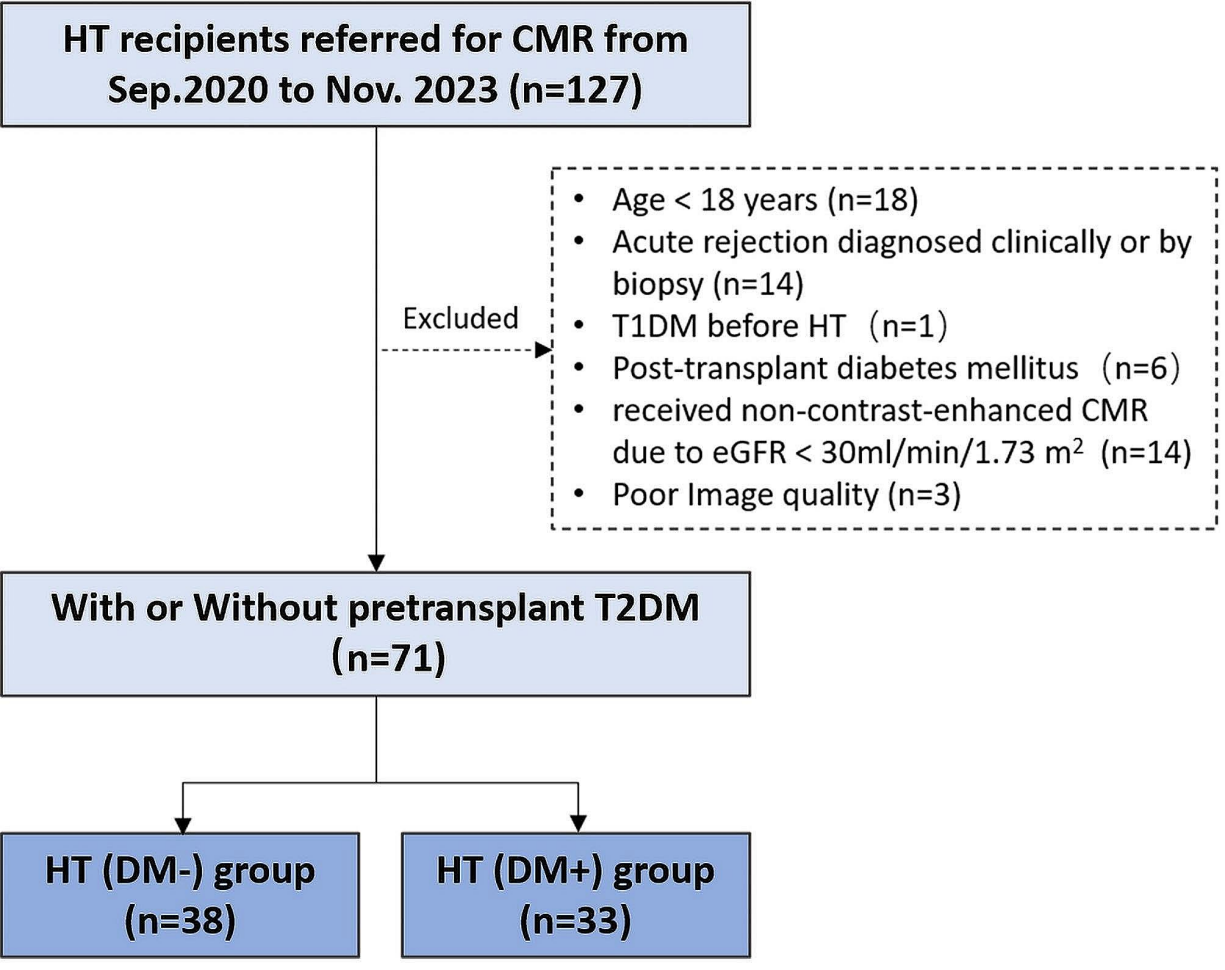
Inclusion and exclusion flowchart for this study. *HT* heart transplantation, *CMR* cardiac magnetic resonance, *T2DM* type 2 diabetes mellitus, *T1DM* type 1 diabetes mellitus, *eGFR* estimated glomerular filtration rate

Since September 2020, CMR examinations have been normalized for all surviving heart transplant recipients at our institute for cardiac surveillance. From September 2020 to November 2023, a total of 127 heart transplant recipients underwent CMR imaging evaluation. The exclusion criteria for the HT recipients (Fig. 1) included age < 18 years (n = 18), previous history of acute rejection (n = 14), pretransplant type 1 diabetes (n = 1), post-transplant diabetes mellitus (n = 6), received non-contrast-enhanced CMR examination due to estimated glomerular filtration rate (eGFR) < 30 mL/min/1.73 m^2^ (n = 14), and poor CMR image quality (n = 3). The remaining 71 patients were then included in this study. The recipients were subsequently categorized into two groups based on the diagnosis of T2DM at the time of HT: recipients with T2DM [HT(DM+)], and recipients without T2DM [HT(DM−)]. A control group of thirty-six healthy individuals (26 males, 10 females) was selected from the database of healthy volunteers, and the same CMR examination was performed. During the CMR scan, demographic information including age, gender, weight, height, and heart rate at rest, was gathered. Data on diabetes course and biochemical markers, including glycated hemoglobin A1c(HbA1c), fasting blood glucose, triglycerides, total cholesterol, high-density lipoprotein (HDL), low-density lipoprotein (LDL), N-terminal pro-brain natriuretic peptide (NT-proBNP), high-sensitivity troponin T (hsTnT), eGFR, and creatinine were collected from medical records prior to CMR examination. To evaluate insulin resistance in heart transplant recipients, we utilized the triglyceride-glucose (TyG) index, which was derived as Ln[fasting triglycerides (mg/dL) × fasting blood glucose (mg/dL)/2] [12]. In addition, diabetes medications (insulin, sodium-glucose cotransporter 2 inhibitors, alpha-glucosidase inhibitors, biguanides, glucagon-like peptide-1/dipeptidyl peptidase-4 inhibitors, sulfonylureas), as well as immunosuppressive medications (tacrolimus, prednisolone, mycophenolic acid, sirolimus) were also collected.

### CMR acquisition

CMR examinations were carried out on clinical 3.0 T magnetic resonance systems (Ingenia, Philips Healthcare). For cine imaging, a balanced steady-state free-precession sequence was employed to acquire short-axis cine images (8- to 12-slice stack), as well as long-axis cine images from 2, 3, and 4 chambers [repetition time/echo time(TR/TE) 3.2/1.60 ms, slice thickness 8 mm, field of view(FOV) 250 × 250 mm^2^, matrix size 168 × 157, flip angle 45°].

For first-pass myocardial perfusion imaging, 0.2 mL/kg gadopentetate dimeglumine was intravenously injected via the cubital vein using an automated injector at a flow rate of 2.5–3.0 mL/s, followed by a 20 mL saline flush. The inversion-recovery echo-planar imaging sequence was used to obtain resting perfusion images from a long-axis 4-chamber view slice and short-axis slices of the basal, midventricular, and apical levels (TR/TE 2.4/1.08 ms, slice thickness 10 mm, FOV 300 × 300 mm, matrix size 100 × 100, flip angle 20°). Ten minutes post-contrast administration, a phase-sensitive inversion recovery (PSIR) sequence was used to acquire late gadolinium enhancement (LGE) images from the short- and long-axis views (TR/TE 3.0/1.50 ms, slice thickness 8 mm, FOV 240 × 240 mm, matrix size 160 × 140, flip angle 45°).

### CMR post-processing

All CMR imaging analyses were conducted using commercial software (cvi42, version 5.17.0; Circle, Calgary, Canada) by two experienced radiologists. The radiologists conducted the analysis in a blinded manner, without access to the clinical data. Conventional CMR parameters were evaluated in the cine images. Firstly, the borders of the LV endo- and epicardium, as well as the right ventricular (RV) endocardium, were delineated semi-automatically on the short-axis using the cvi42 short-3D module at both end-systole and end-diastole. The contours drawn in each phase were verified and manually adjusted as needed to optimize endocardial tracking. The LV geometric and functional parameters, including LV end-systolic volume (LVESV), LV end-diastolic volume (LVEDV), LV mass at end-diastole (LVM), LV ejection fraction (LVEF), cardiac output (CO), and stroke volume, as well as the RV geometric and functional parameters, including RV end-systolic volume (RVESV), RV end-diastolic volume (RVEDV) and RV ejection fraction (RVEF) were then automatically calculated. The Dubois formula was used to index the LVESV, LVEDV, RVESV, RVEDV, and LV mass (LVESVI, LVEDVI, RVESVI, RVEDVI, and LVMI, respectively) based on the body surface area [13]. Additionally, the LV remodeling index, represented by the LVM/LVEDV ratio, was also incorporated into the analysis.

Two-, three-, and four-chamber cine images were selected at end-diastole for evaluation of LV myocardial deformation, and the LV endo- and epicardium contours were semi-automatically outlined with manual correction. The three-dimensional (3D) tissue feature tracking module was utilized to determine the global longitudinal strain (GLS), radial strain (GRS), and circumferential strain (GCS) of the left ventricle. Notably, a positive value for radial strain indicates myocardial thickening, whereas negative values for circumferential and longitudinal deformations reflect myocardial shortening during ventricular wall contraction.

For the assessment of microcirculation perfusion, the endocardial and epicardial contours of the basal, midventricular, and apical short-axis slices were manually delineated on the first-pass perfusion images, along with a region of interest in the LV blood pool. The time-signal intensity curve was then generated, from which semi-quantitative left ventricular perfusion parameters were automatically calculated for each of the 16 myocardial segments. Myocardial perfusion parameters included the time to peak myocardial signal intensity (TTM), maximum signal intensity (MaxSI), and Upslope. To obtain global myocardial perfusion indices, the regional values from the 16 myocardial segments were averaged for each subject. The LGE images were then analyzed by two investigators, who categorized them into three patterns: none, non-infarct, or infarct patterns [14].

### Reproducibility of LV global deformation and perfusion parameters

To evaluate intra-observer variability, the same investigator (LQ.C) randomly selected and re-measured a subset of 20 cases after a 4-week interval. Subsequently, an independent second investigator (C.L) carried out a separate examination of the measures, remaining unaware of the first investigator’s results. Ultimately, inter-observer variability was evaluated based on the findings from both investigators.

### Statistical analysis

All data were analyzed using SPSS statistics software version 26.0, and the statistical graphs were created using GraphPad Prism software version 10.1.0. As appropriate, we used Fisher’s exact test or chi-square test for categorical variables. The normality of the continuous variables was assessed using the Kolmogorov–Smirnov test. Continuous variables following a normal distribution were expressed using the mean ± standard deviation (SD), whereas non-normally distributed data was reported using the median (25–75% interquartile range). Differences among normal controls and HT recipients with or without pretransplant T2DM were assessed using either one-way analysis of variance (ANOVA) or Kruskal–Wallis tests, depending on the normality of the data. Student’s *t*-test and nonparametric tests were used to compare biochemical indicators in HT recipients. To determine the correlation between biochemical indicators and LV strain or perfusion parameters, Spearman’s test was used. Univariable and multivariable linear regression with backward stepwise selection was used to determine the effect of T2DM and perfusion indices on LV strains in HT recipients. The intraclass correlation coefficient (ICC) was used to assess the intra- and interobserver variability of the LV strains and perfusion parameters. The statistical significance of all analyses was determined by p-values < 0.05.

## Results

### Participant characteristics

The present study included a total of 107 participants, consisting of 71 patients and 36 controls. Of all the included HT recipients, 33 had a diagnosis of T2DM prior to the transplantation, and 38 had no history of T2DM. Table 1 summarizes the main clinical characteristics of the HT recipients and controls. In recipients with T2DM prior to transplantation, ischemic cardiomyopathy or coronary artery disease was more likely to be the cause of HT. The median time from heart transplantation to CMR was 12 months in the HT(DM−) group and 11 months in the HT(DM+) group (p = 0.217). Among the three groups, the HT(DM+) group exhibited a higher average age compared to both the control group and HT(DM−) group; BMI and resting heart rate were significantly higher in the HT(DM+) group than in the other two groups (all p < 0.05). In comparison with the HT(DM−) group, the HT(DM+) group demonstrated lower eGFR levels (p < 0.001). As anticipated, the HT(DM+) group exhibited significantly higher fasting blood glucose and HbA1c levels than the HT(DM−) group (both p < 0.001). Additionally, the TyG index in the HT(DM+) group was significantly higher than that in the HT(DM−) group (p < 0.001). The HT(DM+) group exhibited significantly increased levels of triglycerides, total cholesterol, LDL, and NT-proBNP when compared to the HT(DM−) group (all p < 0.05). However, HDL, hsTnT, and creatinine levels did not differ significantly among the HT recipient groups.

**Table 1:** Baseline clinical characteristics of the study cohort

Variables	Controls (n = 36)	HT(DM−) (n = 38)	HT(DM+) (n = 33)	*P* value
Age, years	42.0 (30.3–51.8)	30.5 (23.0–47.3)	53.0 (46.5–59.5)^ab^	** < 0.001**
Male, n (%)	26 (72.2)	27 (73.0)	26 (83.9)	0.452
BMI, kg/m^2^	22.1 ± 1.3	21.3 ± 3.4	23.8 ± 2.9^ab^	**0.001**
Rest heart rate, bpm	69.9 ± 9.8	81.6 ± 13.4^a^	90.1 ± 12.3^ab^	** < 0.001**
Hypertension, n (%)	–	0	6 (19.4)	** < 0.001**
Time from HT to CMR, months	–	12.0 (6.0–13.3)	11.0 (5.0–12.0)	0.217
Donor characteristics				
Donor age, years	–	34.6 ± 10.5	35.6 ± 10.1	0.660
Donor sex, male (%)	–	36 (97.3)	27 (87.0)	0.133
Donor/recipient sex match, n (%)	–	26 (70.3)	26 (83.9)	0.275
Time of ischemia donor heart, min	–	202.5 (170.0–240.0)	178.0 (161.0–203.0)^b^	**0.049**
Laboratory data				
Fasting blood glucose, mmol/L	–	5.3 ± 0.6	6.5 ± 1.1^b^	** < 0.001**
HbA1c, %	–	5.7 (5.5–6.0)	6.6 (6.2–7.7)^b^	** < 0.001**
TyG index		8.48 ± 0.28	9.05 ± 0.63^b^	** < 0.001**
Triglycerides, mmol/L	–	1.2 (0.9–1.4)	1.7 (1.2–2.2)^b^	**0.001**
Total cholesterol, mmol/L	–	4.7 ± 0.9	5.3 ± 1.4^b^	**0.035**
High-density lipoprotein, mmol/L	–	1.3 (1.1–1.5)	1.4 (1.1–1.5)^b^	0.856
Low-density lipoprotein, mmol/L	–	2.9 (2.3–3.3)	3.4 (2.5–4.1)	**0.038**
eGFR, mL/min/1.73 m^2^	–	76.6 ± 20.4	59.2 ± 18.0^b^	** < 0.001**
Creatinine, umol/L	–	93.9 (80.5–111.0)	98.7 (91.8–123.4)	0.090
NT-proBNP, pg/mL	–	254.6 (106.6–411.1)	424.1 (186.4–710.7)^b^	**0.015**
hsTnT, pg/mL	–	13.7 (7.7–29.4)	18.6 (12.1–40.3)	0.079
Immunosuppression, n (%)				
Tacrolimus	–	38 (100.0)	32 (97.0)	0.465
Prednisolone	–	32 (84.2)	33 (100.0)^b^	**0.019**
Mycophenolate mofetil	–	38 (100.0)	33 (100.0)	–
Sirolimus	–	2 (5.3)	3 (9.1)	0.658
Indication for transplant, n (%)				
Dilated cardiomyopathy	–	19 (50.0)	13 (39.4)	0.256
Ischemic heart disease	–	3 (7.9)	15 (45.5)^b^	** < 0.001**
Other	–	16 (42.1)	5 (15.2)^b^	**0.019**
Hospital stay				
Days in ICU	–	5.0 (5.0–8.0)	6.0 (5.0–7.5)	0.583
Days in hospital	–	18.0 (14.0–22.0)	18.0 (15.0–22.0)	0.944
Diabetes treatment, n (%)				
Insulin	–	–	6 (18.2)	–
α‑Glucosidase inhibitors	–	–	4 (12.1)	–
Biguanides	–	–	10 (30.3)	–
SGLT2 inhibitors	–	–	16 (48.5)	–
GLP-1/DPP-4 inhibitors	–	–	13 (39.4)	–
Sulfonylureas	–	–	3 (9.1)	–

Bold indicates statistical significance

*HT* heart transplant, *BMI* body mass index, *CMR* cardiac magnetic resonance, *bpm* beat per minute, *TyG index* triglyceride-glucose index, *ICU* intensive care unit, *SGLT2* sodium-glucose cotransporter 2, *GLP-1/DPP-4* glucagon-like peptide-1/dipeptidyl peptidase-4

^a^HT recipients vs. controls (P < 0.05)

^b^HT recipients with pretransplant T2DM vs. HT recipients without pretransplant T2DM (P < 0.05)

### Comparison of cardiac magnetic resonance-derived parameters among the three groups

As demonstrated in Table 2, the LVEDVI, LVESVI and LVSV in both the HT(DM−) group and HT(DM+) group were significantly lower than those in the control group (all P < 0.05), while they were not significantly different in the HT patient groups (all P > 0.05). No significant differences in LV mass index were detected between HT recipients and controls. HT recipients with pretransplant T2DM had a higher occurrence of non-infarct LGE compared to those without T2DM, although the difference was not statistically significant (51.5% vs. 34.2%, p = 0.157). Regarding the RV geometric and functional parameters, the HT patient groups exhibited a lower RVEDV index and RVESV index than the control group (p < 0.05). Among the three groups, there was no significant difference in RVEF (p > 0.05).

**Table 2:** CMR findings between normal controls and HT recipients with and without pretransplant T2DM

Variables	Controls	HT(DM−)	HT(DM+)	*P* value
LV function parameters				
LVEDVI, mL/m^2^	75.5 (66.3–81.3)	59.8 (54.9–67.3)^a^	54.5 (50.6–58.1)^a^	** < 0.001**
LVESVI, mL/m^2^	25.7 (22.8–30.3)	22.3 (19.4–25.6)^a^	21.6 (19.4–25.1)^a^	**0.001**
LVSV, mL	81.3 ± 14.6	62.7 ± 10.9^a^	56.1 ± 10.6^ab^	** < 0.001**
LVEF, %	63.4 (62.2–65.3)	62.1 (59.0–65.5)	60.0 (53.5–63.2)^a^	**0.001**
LVMI, g	48.0 (40.5–52.8)	47.1 (41.2–53.2)	45.4 (38.0–49.7)	0.401
CO, L/min	5.7 (4.8–6.6)	5.3 (4.6–6.1)	4.7 (4.3–5.5)	**0.007**
LV remodeling index, g/mL	0.63 (0.59–0.68)	0.80 (0.73–0.84)^a^	0.80 (0.70–0.94)^a^	** < 0.001**
RV function parameters				
RVEDVI, mL/m^2^	75.9 ± 15.4	59.6 ± 11.3^a^	55.8 ± 11.8^a^	** < 0.001**
RVESVI, mL/m^2^	35.3 ± 8.9	28.0 ± 6.5^a^	26.9 ± 6.5^a^	** < 0.001**
RVEF, %	52.7 (50.2–56.5)	53.4 (49.7–57.4)	52.0 (48.1–56.6)	0.466
Myocardial strain (%)				
LVGLS	− 17.1 (− 19.2 to − 16.3)	− 16.6 (− 18.5 to − 15.3)	− 13.8 (− 15.5 to − 12.9)^ab^	** < 0.001**
LVGRS	36.6 ± 4.8	36.1 ± 5.1	30.2 ± 6.5^ab^	** < 0.001**
LVGCS	− 20.2 ± 1.6	− 19.6 ± 1.6	− 17.5 ± 2.5^ab^	** < 0.001**
Myocardial perfusion				
TTM, s	26.1 ± 2.6	26.8 ± 4.1	31.0 ± 5.9^ab^	** < 0.001**
MaxSI	330.9 (291.3–372.6)	316.1 (283.3–365.8)	328.8 (286.6–393.6)	0.701
Upslope	36.9 (31.3–45.6)	35.2 (28.2–41.6)	36.6 (32.2–42.2)	0.618

Bold indicates statistical significance

*LV* left ventricular, *RV* right ventricular, *EDVI* end-diastolic volume index, *ESVI* end-systolic volume index, *EF* ejection fraction, *SV* stroke volume, *MI* mass index, *CO* cardiac output, *GLS* global longitudinal strain, *GRS* global radial strain, *GCS* global circumferential strain, *TTM* time to peak myocardial signal intensity, *MaxSI* maximum signal intensity

^a^HT recipients vs. controls (P < 0.05)

^b^HT recipients with pretransplant T2DM vs. HT recipients without pretransplant T2DM (P < 0.05)

**Fig. 2:**
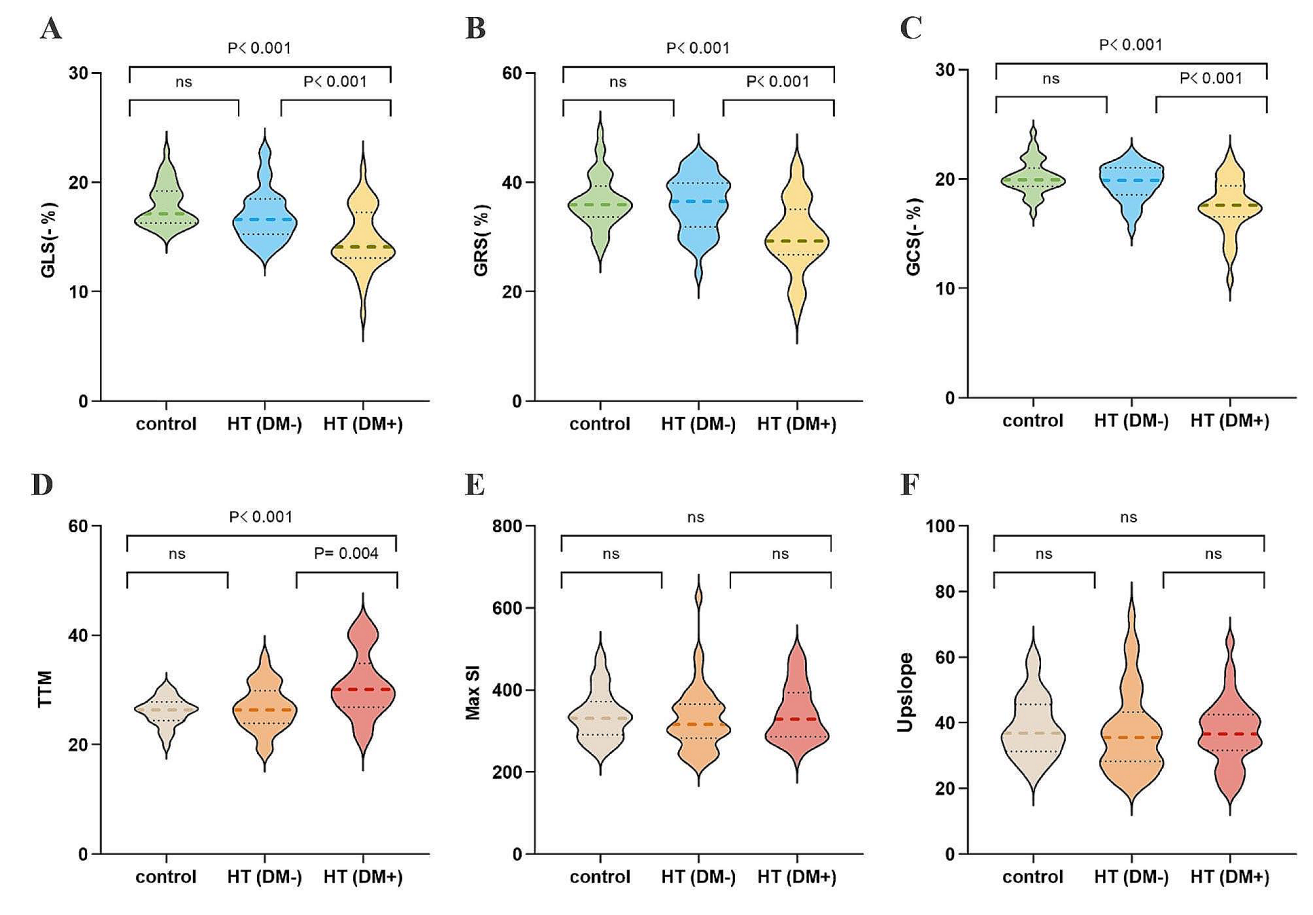
Comparative analysis of LV global strains (**A**–**C**) and perfusion parameters (**D**–**F**) between controls, HT recipients without pretransplant T2DM [HT(DM−)], and HT recipients with pretransplant T2DM [HT(DM+)]. *GLS* global longitudinal strain, *GRS* global radial strain, *GCS* global circumferential strain, *TTM* time to peak myocardial signal intensity, *MaxSI* maximum signal intensity. For avoiding the influence of directional sign, the absolute values of the GLS and GCS for the LV were used. *ns* P > 0.05

For LV global myocardial strains, the GLS, GRS and GCS of the HT(DM+) group were significantly lower than those of the HT(DM−) group and control group (longitudinal: − 13.8% (− 15.5, − 12.9) vs. − 16.6% (− 18.5, − 15.3) vs. − 17.1% (− 19.2, − 16.3); radial: 30.2% ± 6.5 vs. 36.1% ± 5.1 vs. 36.6% ± 4.8; circumferential: − 17.5% ± 2.5 vs. − 19.6% ± 1.6 vs. 20.2% ± 1.6; all p < 0.05; Fig. 2), whereas the latter two groups exhibited no statistically significant difference (all p > 0.05).

**Fig. 3:**
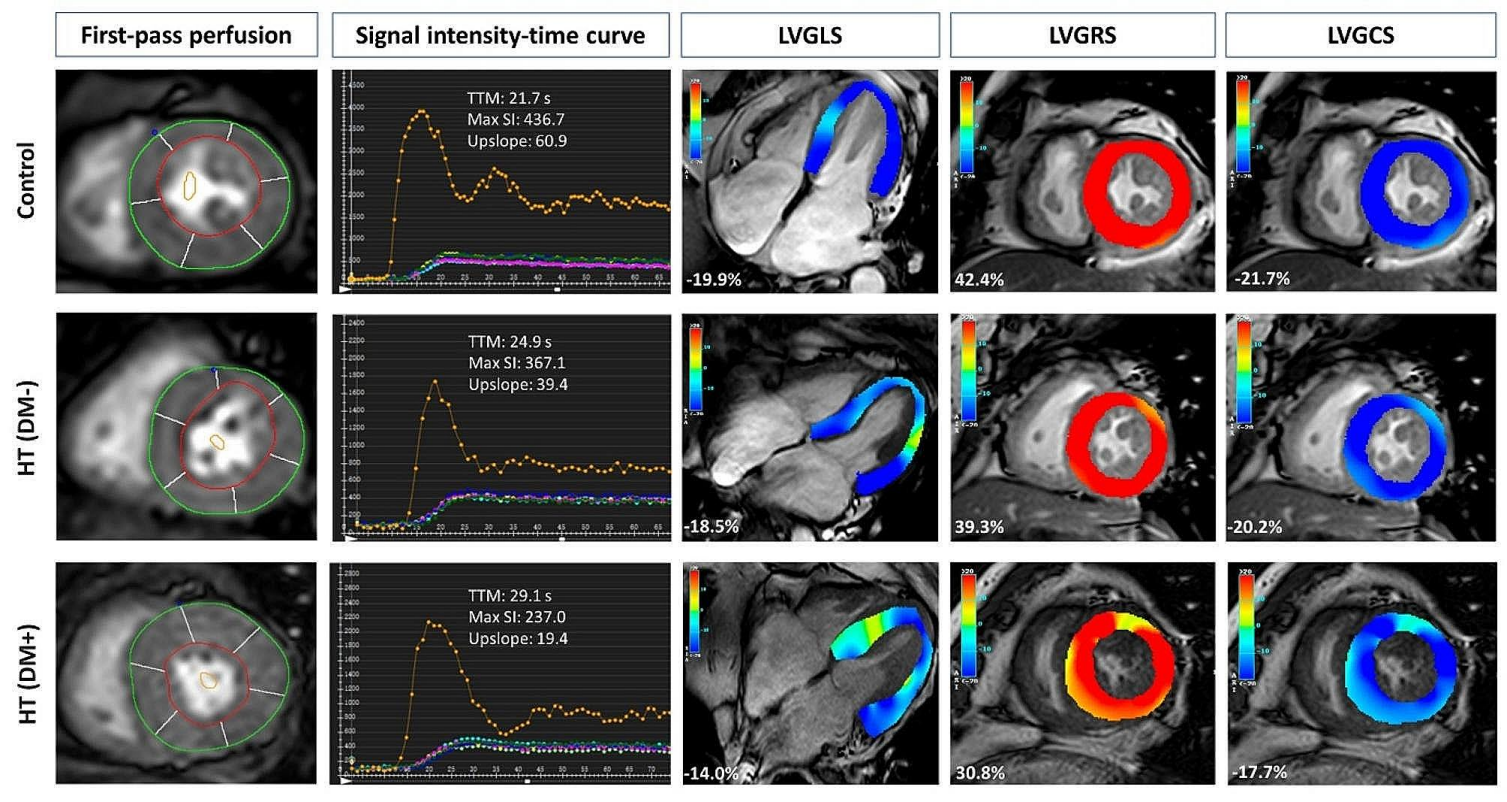
Representative first-pass perfusion images (first column), time‐signal intensity curves (second column) from the mid‐left ventricular slice, and pseudo-color plots of left ventricular global longitudinal, radial, and circumferential strain (third, fourth, and fifth column respectively) are presented for a normal control (top row), a heart transplant recipient without pretransplant T2DM (second row), and a heart transplant recipient with pretransplant T2DM (bottom row)

Table 2 presents the first-pass perfusion parameters for all subjects. The HT(DM+) group exhibited a significantly higher TTM (31.0 ± 5.9 vs. 26.8 ± 4.1 vs. 26.1 ± 2.6, p < 0.001) in comparison to the other two groups, but there was no significant difference between the HT(DM−) group and the control group (p > 0.05). There was no significant difference between the observed groups in Upslope or MaxSI (all p > 0.05). The representative CMR first-pass perfusion and global strain images in a control, an HT patient without pretransplant T2DM, and an HT patient with pretransplant T2DM are illustrated in Fig. 3.

### Associations between CMR-derived indices and biochemical indicators in all HT recipients

Table 3 lists the correlations between biochemical indicators and myocardial perfusion indices or LV strains. Spearman correlations revealed that HbA1c was inversely correlated with the magnitude of strain (r = − 0.399 for longitudinal strain, − 0.362 for radial strain, and − 0.389 for circumferential strain; all P < 0.05), while it was positively correlated with the TTM (r = 0.485, P < 0.001) among all HT recipients in this cohort. The TyG index showed a negative correlation with the magnitude of LV strains and a positive correlation with the TTM. Furthermore, fasting plasma glucose and eGFR were also significantly associated with the magnitude of LV strains and TTM (all P < 0.05). Moreover, we conducted univariate and multivariate linear regression analyses (Table 4) to assess the independent impact of T2DM on LV global strains in HT recipients. When incorporating both T2DM and perfusion indices into the regression models, T2DM was found to have an independent association with LV global strains (β = − 0.508, longitudinal; − 0.370, radial; − 0.371, circumferential) (all P < 0.05).

**Table 3:** Correlations between LV strains or first-pass myocardial perfusion parameters and biochemical indicators in HT recipients

	GLS		GRS		GCS		TTM		Max SI		Upslope	
	R	P	R	P	R	P	R	P	R	P	R	P
HbA1c	− 0.399	**0.001**	− 0.362	**0.002**	− 0.389	**0.001**	0.485	** < 0.001**	0.014	0.910	− 0.131	0.276
Fasting plasma glucose	− 0.278	**0.019**	− 0.317	**0.007**	− 0.307	**0.009**	0.284	**0.016**	0.018	0.883	− 0.048	0.692
TyG index	− 0.428	** < 0.001**	− 0.321	**0.006**	− 0.299	**0.011**	0.296	**0.012**	0.084	0.484	0.137	0.255
eGFR	0.284	**0.016**	0.368	**0.002**	0.387	**0.001**	− 0.341	**0.004**	0.049	0.687	0.118	0.328
NT-proBNP	− 0.136	0.260	− 0.368	**0.002**	− 0.353	**0.003**	0.146	0.226	0.031	0.796	0.044	0.715
Triglycerides	− 0.377	**0.001**	− 0.208	0.082	− 0.199	0.096	0.243	**0.041**	0.050	0.676	0.136	0.257
Total cholesterol	− 0.110	0.360	− 0.023	0.847	0.006	0.962	− 0.076	0.527	0.196	0.102	0.191	0.110

Bold indicates statistical significance

*TyG index* triglyceride-glucose index

**Table 4:** Univariable and multivariable analysis between the LV strain and pretransplant T2DM in all HT recipients adjusted for age, sex, BMI, resting heart rate, eGFR, and perfusion indices

	GLS			GRS			GCS		
	Univariable	Multivariable		Univariable	Multivariable		Univariable	Multivariable	
	r	β	p	r	β	p	r	β	p
Pretransplant T2DM	− 0.508*	− **0.508**	** < 0.001**	− 0.459*	− **0.371**	**0.002**	− 0.456*	− **0.370**	**0.002**
Age	− 0.186	–	–	− 0.311*	0.036	0.799	− 0.364*	− 0.064	0.658
Sex	0.219*	0.152	0.147	0.077	–	–	0.086	–	–
BMI	0.012	–	–	− 0.111	–	–	0.120	–	–
Heart rate	− 0.260*	− 0.101	0.354	− 0.126	–		− 0.124	–	–
eGFR	0.295*	0.086	0.454	0.364*	0.211	0.072	0.361*	0.208	0.077
TTM	− 0.078	–	–	− 0.128	–	–	− 0.159	–	–
Upslope	− 0.033	–		− 0.051	–	–	0.001	–	–
MaxSI	− 0.147	–	–	− 0.142	–	–	− 0.089	–	–

Bold indicates statistical significance

*p < 0.1

### Inter- and intraobserver consistency tests

The inter- and intraobserver agreement were examined for LV deformation and first-pass myocardial perfusion indices. The ICCs for inter- and intraobserver variabilities were found to be 0.900–0.948 and 0.948–0.982, respectively, for LV deformation; for first-pass myocardial perfusion, the ICCs were 0.914–0.963 and 0.937–0.982, respectively (Supplementary File: Table S1).

## Discussion

By utilizing CMR imaging, this study examined the features of LV strains and perfusion in HT recipients with or without pretransplant T2DM. The major findings of this study are as follows: (1) we verified the occurrence of decreased left ventricular strains among HT recipients with pretransplant T2DM in comparison to those without pretransplant T2DM and normal individuals; (2) among the perfusion indices studied, TTM was increased in the HT(DM+) group, while there were no significant differences in Upslope or MaxSI among the groups; and (3) finally, T2DM was an independent determinant of LV strains. Our research indicated that T2DM adversely impacts myocardial systolic function and may even impair myocardial perfusion in heart transplant recipients, which may lead to heightened cardiovascular risk.

In recent years, there has been a noticeable increase in the incidence of diabetes among HT recipients. According to data from the United States National Registry, the prevalence of pretransplant T2DM increased from 22.2% in 2006 to 27.9% in 2021 [5]. This finding contrasts with earlier studies from previous eras of heart transplantation, where the prevalence of pretransplant diabetes was considerably lower, ranging between 13.7 and 18.3% [15, 16]. The increasing prevalence of diabetes among HT recipients in the coming decades may result in an increase in mortality [5, 17]. Previous research has demonstrated that subclinical LV systolic dysfunction is independently linked to all-cause death in T2DM patients [18]. HT recipients with pretransplant T2DM may benefit from early detection of myocardial dysfunction, as this allows for the recommendation of targeted treatment strategies aimed at mitigating this progression, ultimately leading to long-lasting advantages in terms of decreasing morbidity and mortality [19, 20]. The presence of microvascular endothelial inflammation, rarefaction and end-product accumulation in diabetic patients can lead to impaired microvascular function, which in turn affects cardiac contractility [21]. Furthermore, prior studies have revealed a correlation between compromised myocardial perfusion and LV dysfunction in patients with T2DM [10, 21]. Therefore, our study aimed to investigate the impact of pretransplant T2DM on myocardial function and microcirculation perfusion in HT recipients, especially in light of the increasing prevalence of T2DM among HT patients.

Our study revealed that T2DM had a deleterious effect on LV systolic dysfunction, as indicated by reduced LV global strains in the HT (DM+) group, despite comparable LVEF and LV geometries found in the HT subgroups. It reflected that myocardial strain, compared to traditional LV function parameters, can more sensitively and promptly detect subclinical LV dysfunction, aligning with previous research [10]. Moreover, we found that the presence of pretransplant T2DM was a significant and independent predictor of left ventricular strains. A large cohort research involving 152 HT recipients revealed that CMR-GLS was independently associated with major adverse cardiac events and long-term mortality [22]. This finding emphasizes the critical need of promptly detecting subclinical myocardial dysfunction in HT recipients with pretransplant T2DM for mitigating cardiovascular disease risk and improving outcomes. The administration of immunosuppressive drugs, including corticosteroids, following heart transplantation can impact glucose homeostasis in transplant recipients, potentially precipitating the onset and progression of diabetes [23, 24].In the HT(DM+) group, all individuals (n = 33) were on prednisolone at a higher rate compared to the HT(DM−) group (100% vs 84.2%), as 29 individuals (87.9%) had not reached the 12-month post-heart transplantation timeframe for prednisolone withdrawal at our institute during the CMR examination. Additionally, 3 (9.1%) recipients in the HT(DM+) group were in the process of reducing prednisolone doses but had not yet fully withdrawn, and 1 (3.0%) recipient had a positive history of donor-specific antibodies. Considering that transplant patients typically require the use of immunosuppressive medication, it is important to monitor the development and progression of diabetes in these patients in order to provide appropriate treatment guidance.

The exact mechanism underlying the reduced strain in HT recipients with pretransplant T2DM is not fully understood. The pathophysiology of T2DM−induced cardiac dysfunction involves mechanisms such as hyperglycemia and calcium disturbances in cardiomyocytes, ultimately resulting in cardiometabolic abnormalities. Studies have indicated that hyperglycemia can promote fatty acid oxidation and induce perivascular and interstitial fibrosis, thereby reducing ventricular compliance [25]. Previous research has also confirmed the association of diabetes with increased myocardial triglyceride content, with a negative correlation observed between epicardial fat and peak systolic strain [26]. Furthermore, the presence of myocardial fat infiltration associated with diabetes has been verified in HT recipients. Based on the analysis of serial cardiac biopsies from 158 HT recipients, Marfella et al. demonstrated that myocardial lipid triglyceride accumulation begins within 3 months post-HT in recipients with pretransplant T2DM, and this accumulation of myocardial lipids was associated with impaired cardiac function [8]. Moreover, hyperglycemia may affect oxidative phosphorylation of myocardial cells in individuals with diabetes. A study on adult heart transplant recipients who received hearts from donors without T2DM revealed that exposure to T2DM can impact the respiratory function of mitochondria in the human ventricular myocardium, despite the absence of structural or coronary heart disease [27]. The findings above suggest that diabetes could impact cardiac contractile function in HT recipients through its influence on cardiomyocyte metabolism. Our previous research has confirmed a gradual recovery of transplantation-related myocardial injury within the initial 12 months post-HT, with GCS and GRS being normalized at 12 months after the procedure [28]. However, the results of this study showed that recipients with pretransplant T2DM had significantly lower LV global myocardial strains compared to those without T2DM. These results imply that HT recipients with T2DM may experience impaired recovery of cardiac function following heart transplantation, potentially attributed to diabetes-related cardiometabolic abnormalities.

The presence of diabetes could potentially impact myocardial perfusion in HT recipients. T2DM has been theorized to contribute to the development of CAV [29], which is a common complication and a significant contributor to mortality among HT recipients [30]. CAV is characterized by diffuse panarteritis, affecting the epicardial coronary arteries as well as the coronary microvasculature [31]. Both CAV and T2DM can manifest structural and functional alterations at the microvascular level in the coronary artery system, including hypertrophy of small coronary arteries and arterioles, microvascular rarefaction, and abnormal vasomotor function induced by endothelial dysfunction [9, 32].

TTM was significantly higher in comparison to both the control group and the HT(DM−) group. However, no significant differences were observed in MaxSI or upslope, suggesting that myocardial perfusion may be compromised, although not significantly, in recipients with pretransplant T2DM. Several potential explanations may account for this observation. First, more than 75% of the HT recipients included in our study underwent CMR within the first 12 months post-HT, suggesting that the impact of diabetes on the transplanted heart may still be in its early stages. Diabetic patients often experience coronary microvascular dysfunction, characterized by early changes in the vasomotion of the coronary arterioles and long-term structural alterations [9]. Typically, anatomical changes in the microvasculature take several years to manifest [9]. Therefore, the impact of diabetes on the transplanted heart in our study may present as vascular vasomotor dysfunction rather than irreversible structural damage such as intimal hyperplasia and vascular remodeling. Second, the maintenance of Upslope and MaxSI in the HT(DM+) group may indicate an adaptive mechanism. Resting myocardial perfusion, which mirrors auto-regulated blood flow, is intricately tied to myocardial oxygen consumption and is predominantly governed by heart rate and the contractility of the myocardium [33]. In comparison to those in the HT(DM−) group, recipients with pretransplant T2DM exhibited a higher heart rate. Consequently, the preserved upslope and MaxSI may be attributed to increased heart rate that helps maintain oxygen supply under conditions of coronary microvascular dysfunction.

Moreover, we found a correlation between TTM and HbA1c levels, suggesting that glycemic control may influence the microvasculature. The primary etiology of coronary microvascular dysfunction is likely to be endothelial dysfunction associated with diabetes, which can be attributed to alterations induced by hyperglycemia [9]. Endothelial cells are particularly susceptible to damage from hyperglycemia compared to other cell types [34]. Under hyperglycemic stress, endothelial cell mitochondria reduce the oxidation of glucose and enhance fatty acid metabolism, leading to a decrease in the ATP/ADP ratio and oxygen consumption [35]. Furthermore, the TyG index is increasingly being recognized as a reliable alternative biomarker for insulin resistance, with an elevated TyG index linked to the development of microvascular complications [36, 37]. Correlation analysis revealed a positive association between the TyG index and TTM, indicating that insulin resistance may contribute to microvascular dysfunction in HT recipients. Effective diabetes management has been demonstrated to improve diabetes-associated endothelial dysfunction prior to the onset of macrovascular complications [35]. Additionally, metformin therapy in HT recipients with T2DM has been found to significantly reduce the long-term risk of CAV and cardiovascular mortality after HT [19]. Hence, timely management of glycemia in patients with pretransplant T2DM could potentially correct microvascular dysfunction. However, in cases where microvascular dysfunction is severe or coexists with other heart conditions, irreversible impairment may persist, leading to a potentially worse prognosis. Therefore, vigilantly monitoring and promptly identifying microcirculation dysfunction may be pivotal for improving the survival and prognosis of HT recipients with pretransplant T2DM.

## Limitations

We recognize several potential limitations in our research. Initially, this investigation was carried out at a single center with a comparatively limited number of participants, highlighting the need for further multicenter research encompassing a broader population to validate our results. Second, it remains unclear whether changes in LV strain lead to cardiovascular events. The HT recipients in our study will be followed up prospectively to explore whether left ventricular strain can be a predictive factor for cardiovascular outcomes in HT recipients with pretransplant T2DM. Third, analogous to the strain obtained from echocardiography, there are algorithmic variations among different CMR-FT strain applications, potentially resulting in different values. However, the assessment of LV myocardial deformation in our study strictly adhered to established guidelines. Finally, fasting blood glucose and lipid profile measurements were not performed on the control group. To verify that the enrolled controls satisfied the study’s inclusion requirements, we carefully reviewed their comprehensive medical records and examination data.

## Conclusion

The presence of T2DM in HT recipients adversely affects left ventricular systolic function and may also impact myocardial perfusion post-heart transplantation. Therefore, it is important to place greater emphasis on the evaluation of LV strain and perfusion in HT recipients with pretransplant T2DM. Considering that this study was conducted in a single-center design with a relatively small number of participants, larger-scale studies are warranted to validate these findings.

### Supplementary Information

Supplementary File 1

## Data Availability

The datasets generated and analyzed during the current study are available from the corresponding authors on reasonable request.

## Abbreviations

T2DM: Type 2 diabetes mellitus
LV: Left ventricular
HT: Heart transplant
ISHLT: International society for heart and lung transplantation
CMR: Cardiovascular magnetic resonance
GLS: Global longitudinal strain
GRS: Global radial strain
GCS: Global circumferential strain
FT: Feature tracking
TyG: Triglyceride-glucose
CAV: Cardiac allograft vasculopathy
EDV: End-diastolic volume
ESV: End-systolic volume
LVEF: Left ventricular ejection fraction
LVMI: LV mass index
TTM: Time to peak myocardial signal intensity
MaxSI: Maximum signal intensity
