# Supplementary material for: Impact of pretransplant T2DM on left ventricular deformation and myocardial perfusion in heart transplanted recipients: a 3.0 T cardiac magnetic resonance study
Source: Cardiovasc Diabetol. 2024 Jun 21;23:216. doi: 10.1186/s12933-024-02323-x (PMC11193171; doi:10.1186/s12933-024-02323-x)
Supplement: Supplementary file 1 [file 12933_2024_2323_MOESM1_ESM.docx]

**Table S1** Intra- and interobserver consistency tests for myocardial strains and perfusion parameters

|  | Intra-observer | |  | Inter-observer | |
| --- | --- | --- | --- | --- | --- |
|  | ICC | 95% CI |  | ICC | 95% CI |
| LV GLS | 0.982 | 0.956-0.993 |  | 0.948 | 0.873-0.979 |
| LV GRS | 0.948 | 0.873-0.979 |  | 0.930 | 0.832-0.972 |
| LV GCS | 0.954 | 0.889-0.982 |  | 0.900 | 0.766-0.959 |
| MaxSI | 0.982 | 0.956-0.993 |  | 0.963 | 0.910-0.985 |
| Upslope | 0.937 | 0.847-0.974 |  | 0.914 | 0.796-0.965 |
| TTM | 0.936 | 0.845-0.974 |  | 0.922 | 0.814-0.968 |
